# Modulation of β-amyloid precursor protein trafficking and processing by the low density lipoprotein receptor family

**DOI:** 10.1186/1750-1326-1-8

**Published:** 2006-08-18

**Authors:** Judy A Cam, Guojun Bu

**Affiliations:** 1 Departments of Pediatrics, and Cell Biology & Physiology, Washington University School of Medicine, St. Louis, Missouri 63110, USA; 2 Department of Pathology, New York University, 550 1^st ^Avenue, New York, New York 10016, USA

## Abstract

Amyloid-β peptide (Aβ) accumulation in the brain is an early, toxic event in the pathogenesis of Alzheimer's disease (AD). Aβ is produced by proteolytic processing of a transmembrane protein, β-amyloid precursor protein (APP), by β- and γ-secretases. Mounting evidence has demonstrated that alterations in APP cellular trafficking and localization directly impact its processing to Aβ. Recent studies have shown that members of the low-density lipoprotein receptor family, including LRP, LRP1B, SorLA/LR11, and apolipoprotein E (apoE) receptor 2, interact with APP and regulate its endocytic trafficking. Another common feature of these receptors is their ability to bind apoE, which exists in three isoforms in humans and the presence of the ε4 allele represents a genetic risk factor for AD. In this review, we summarize the current understanding of the function of these apoE receptors with a focus on their role in APP trafficking and processing. Knowledge of the interactions between these distinct low-density lipoprotein receptor family members and APP may ultimately influence future therapies for AD.

## Background

Alzheimer's disease (AD) is the most common cause of dementia among people age 65 and older. A diagnosis of AD is confirmed upon autopsy by the presence of characteristic lesions in specific regions of the brain, notably the hippocampus, amygdala, and association cortices of the frontal, temporal and parietal lobe of the cortex [1]. Fittingly, these affected regions are responsible for memory, emotion and decision making abilities, which are impaired in AD dementia. Lesions found in AD are deposits of amyloid plaques in the cerebrovasculature and parenchyma of the brain and intracellular neurofibrillary tangles. Amyloid plaques are either dense/fibrillar or diffuse in nature; fibrillar plaques are surrounded by dystrophic neurites, activated microglia, and reactive astrocytes, while diffuse plaques lack fibrils and are associated with few or no dystrophic neurites or altered glia.

A major component of the amyloid plaques found in AD is the ~4 kDa amyloid-β peptide (Aβ) [2], which is a cleavage product of the β-amyloid precursor protein (APP) [3]. Aβ ranges in size from 37 to 43 amino acids; however, Aβ42(43) may act as a pathogenic seed for fibrillar plaque formation since it is found in insoluble cores of fibrillar and diffuse plaques [4]. One current hypothesis known as the "amyloid hypothesis" postulates that increased Aβ production or reduced Aβ metabolism results in the formation of aggregated Aβ deposits leading to AD dementia (for review see [5]). In support of this idea, *in vitro *studies have demonstrated that Aβ42 aggregates and forms fibrils more rapidly and is more neurotoxic than Aβ40 [6-8]. *In vivo*, studies in mice demonstrate that expression of only human Aβ42 not Aβ40 results in overt amyloid pathology indicating a requirement for Aβ42 in Aβ plaque deposition and AD pathogenesis [9]. It is possible that aggregation of Aβ into fibrils is not the principal cause of AD dementia. Recent studies have also associated non-fibrillar assemblies of Aβ with neuronal injury, synaptic loss and dementia associated with AD. These Aβ assemblies, including soluble Aβ oligomers and intraneuronal Aβ deposits, have been hypothesized to act as an early, causal factor in the pathogenesis AD [1,10].

Genetic studies have confirmed that the processing of APP to Aβ is important for AD pathogenesis. Mapping of genes that segregate within families that develop early onset AD dementia (<65 years of age) led to the identification of a mutation in the *APP *gene on chromosome 21 [11]. Twenty-five separate pathogenic mutations within the *APP *gene have been described in familial cases of AD [12]. Several of these mutations increase APP processing to Aβ. Furthermore, persons affected by Down's syndrome (trisomy-21), who have three copies of chromosome 21 and therefore the *APP *gene, inevitably develop AD. Individuals who have Down's syndrome but lack the region of chromosome 21 containing the *APP *gene do not develop AD [13]. Together, these findings imply that a gain-of-function mechanism for APP is an important factor in the development of AD. Although genetic mutations in *APP*, have enhanced our understanding of the biology of AD, they only account for <1% of known AD cases [12]. For this reason, it is of interest to study proteins that interact with APP and modulate its processing to Aβ.

## APP biology and processing

The APP gene is alternatively spliced to produce three major isoforms of 695, 751, and 770 amino acids in length. The two longer APP isoforms, APP751 and APP770, both contain a 56 amino acid Kunitz Protease Inhibitor (KPI) homology domain within their extracellular regions. APP is ubiquitously expressed throughout the body, but APP695, which lacks the KPI domain, is the predominant form found in neurons [14,15], and may play a role in neurite outgrowth and axonal sprouting (for review see [16]). Targeted deletion of the *APP *gene in mice produces no apparent phenotype, suggesting that other members of the APP family, such as amyloid precursor like proteins-1 (APLP1) and 2 (APLP2), can compensate for its function [17]. Triple knockout mice lacking APP, APLP1 and APLP2 die shortly after birth and have cranial abnormalities or cortical dysplasia, suggesting an essential function of this gene family in neuronal migration and brain development [18]. APP and APLP2 may have functional redundancy in development since a double knockout mouse of both genes displays a post-natal lethal phenotype while a double knockout mouse of APP and APLP1 is viable [19]. APP, however, is the only member of the family to contain the Aβ region and produce the AD-associated Aβ peptide.

**Figure 1:**
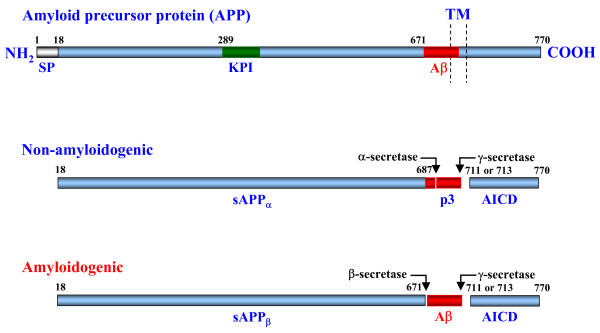
**Schematic representation of APP processing. **APP is a type I transmembrane protein that can undergo two separate proteolytic pathways. In the non-amyloidogenic pathway, APP is processed by an α-secretase that clips within the Aβ region (thus precluding its formation), resulting in the release of a soluble ~110–120 kDa N-terminal APP fragment (sAPPα). This pathway also releases a CTF that is 83 amino acids in length (C83). C83 can also be cleaved by a γ-secretase to release a small, non-toxic 3 kDa fragment known as p3 and a γCTF known as APP intracellular domain (AICD). In the amyloidogenic pathway, APP is cleaved first by β-secretase releasing a sAPPβ fragment and leaving a 99 amino acid CTF attached to the membrane (C99). C99 is subsequently cleaved by a γ-secretase, within its intramembrane region to release the Aβ peptide and AICD. SP, Signal Peptide; KPI, Kunitz-type Proteinase Inhibitor domain.

During its trafficking to the cell surface and in the endocytic pathway, APP can undergo proteolysis by secretase enzymes to release either the Aβ peptide (amyloidogenic pathway) or a shorter, non-toxic peptide known as sAPP (non-amyloidogenic pathway) [20] (Fig. 1). In the amyloidogenic pathway, APP is first cleaved at a β-secretase site by the enzyme BACE (β-site APP cleaving enzyme), which releases a soluble β-cleaved APP fragment (sAPPβ) and leaves a 99 amino acid C-terminal fragment (CTF) known as C99 attached to the membrane. C99 is subsequently cleaved by a γ-secretase/presenilin complex within its intramembrane region to release the Aβ peptide [1]. In the non-amyloidogenic pathway, APP is processed by an α-secretase that clips within the Aβ region, which results in the release of a soluble ~110–120 kDa α-cleaved APP fragment (sAPPα). This pathway also releases a CTF that is 83 amino acids in length known as C83. C83 can also be cleaved by γ-secretase to release p3. In both the amyloidogenic and non-amyloidogenic pathway, the γ-secretase cleavage of APP can also release an APP intracellular domain fragment (AICD). The processing of APP to these separate components may have important consequences in both diseased and normal physiology (for review see [21]). sAPP, which contains a KPI domain, has been identified as the serine protease inhibitor, protease nexin II (PNII), which inhibits the serine protease factor XIa in the blood coagulation cascade [22,23]. In addition, the C-terminal cleavage products of APP may activate gene transcription in concert with other proteins such as FE65 [24].

## Endocytic trafficking of APP

The presence of APP and APP cleavage products in clathrin-coated vesicles first suggested that the amyloidogenic processing of APP could occur in the endocytic pathway [25]. In 1994, Koo and Squazzo showed that cell surface radiolabeled APP releases Aβ, and that endocytosis of APP is also necessary for Aβ production. Inhibiting endocytosis of cell surface APP by potassium depletion, which disrupts the formation of clathrin lattices, or by C-terminal deletions of the APP tail, which removes important internalization motifs, leads to a decrease in Aβ production along with an increase in cell surface APP and sAPPα secretion [26].

The cytoplasmic tail of APP contains two motifs, YENPTY and YTSI, which are similar to the tyrosine-based NPXY and YXXØ (where X can be any amino acid and Ø is any amino acid with a bulky hydrophobic group) consensus endocytic motifs found in other well-known endocytic receptors such as the low-density lipoprotein and epidermal growth factor receptors [27]. Fusing the APP tail to the ectodomain of the transferrin receptor resulted in a functional endocytic receptor [28]. However, the endocytosis rate mediated by the APP tail is relatively slow at ~6%/minute [29]. Using metabolic labeling followed by cell surface biotinylation, Lai *et al*. (1995) demonstrated that deletion of the YENPTY motif in full length APP both decreased endocytosis and increased sAPPα secretion. Using a radioiodinated monoclonal antibody against APP to monitor its trafficking, it was also shown that deletion of the entire APP tail increased APP retention at the cell surface and sAPPα production by 2.5-fold when compared to wild-type APP [30]. Further mutational analyses indicated that the dominant endocytosis motif within the APP tail was the tetrapeptide YENP [31].

These studies establish a tight correlation between APP endocytosis and Aβ secretion. Substantial research efforts have examined the localization of the secretases involved in APP processing. The recent identification of the endosomally localized β-secretase, BACE, further supports the idea that Aβ is formed in the endocytic pathway. BACE localizes to the Golgi and endosomes and has optimal activity at the acidic pH found within endosomal compartments [32]. Components of the γ-secretase complex have been localized to the endoplasmic reticulum (ER), lysosomes and cell surface [33,34], whereas α-secretase activity is found primarily at the cell surface [35]. Since the secretases responsible for APP proteolysis have optimal enzymatic activity or distribution within specific cellular compartments, shifting APP to these compartments leads to an increased probability that APP will be cleaved by that secretase.

When cell surface APP is internalized to the endosomes, it is cleaved at the β-secretase site by BACE, and then returned to the cell surface or trafficked to the lysosome where it is cleaved by γ-secretase to produce Aβ. On the other hand, if APP accumulates at the cell surface it has a greater availability for α-secretase interaction and is cleaved to sAPPα via the non-amyloidogenic pathway. Although a large amount of work has been devoted to the study of APP trafficking within the endocytic pathway, there is only emerging evidence that APP-interacting receptors can affect its trafficking and processing. This review focuses on several members of the low density lipoprotein receptor family that have been shown to interact and influence the cellular localization and processing of APP to Aβ.

## The low density lipoprotein (LDL) receptor family

**Figure 2:**
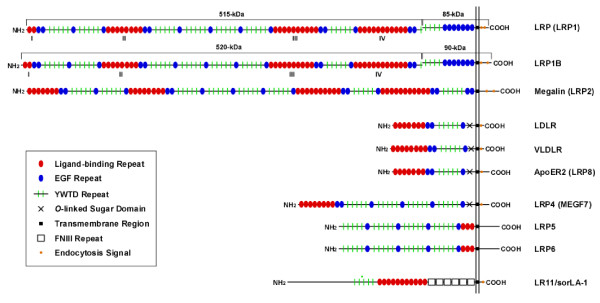
**Schematic representation of members of the LDLR family. **Members of the LDLR family have diverse functions from cholesterol metabolism, Reelin and Wnt signalling, to intracellular transport. Despite multiple functions, they share common structural motifs, including ligand-binding repeats, epidermal growth factor (EGF) repeats, YWTD spacer domains, a single transmembrane domain and a short cytoplasmic domain containing conserved endocytic motifs. FN, fibronectin.

The LDL receptor family consists of a large class of cell surface receptors of diverse function. The family currently consists of the LDL receptor, LDL receptor-related protein (LRP, also known as LRP1), LDL receptor-related protein 1B (LRP1B), megalin/LRP2, the very low density lipoprotein receptor (VLDLR), apoE receptor 2 (apoER2), LRP4/MEGF7, LRP5, LRP6, and sorting protein-related receptor containing LDLR class A repeats (sorLA) or LR11 (Fig. 2). Although members of the LDL receptor family perform a variety of functions from cholesterol metabolism to cellular signaling, they share several features and structural motifs: 1) ligand-binding complement-type cysteine-rich repeats, 2) epidermal growth factor (EGF) receptor-like repeats, 3) YWTD β-propeller domains, 4) one or more endocytic motifs within their cytoplasmic domains such as NPXY, YXXØ or di-leucine motifs [36], and 5) binding of apolipoprotein E (apoE), a protein involved in cholesterol transport. Interestingly, apoE exists in three isoforms (E2, E3, and E4) in humans, and the presence of an ε4 allele represents a genetic risk factor for late-onset AD [37].

## LRP

LRP is a multi-functional endocytic receptor that is highly expressed in the brain. At ~600 kDa in size, LRP is one of the largest receptors of the LDL receptor family. LRP is synthesized as a single polypeptide precursor and is cleaved by furin in the *trans*-Golgi network to produce a non-covalently associated heterodimer: a heavy chain (~515 kDa) containing the extracellular and ligand binding domains of LRP and a light chain (85 kDa) containing the transmembrane domain and cytoplasmic tail of LRP. The 515 kDa subunit contains four putative ligand binding domains (designated by Roman numerals I, II, III, and IV) [38] consisting of 2, 8, 10 and 11 cysteine-rich complement-type repeats, respectively. Each of these clusters is interspersed with EGF precursor homology repeats and YWTD repeats that form β-propeller modules (Fig. 2).

Two approaches have been successfully employed to identify which cluster of ligand binding repeats within LRP is responsible for binding to ligands. In one approach, LRP minireceptors were generated by fusing various clusters of ligand-binding repeats to the LRP light-chain and measuring their ability to mediate ligand internalization [39,40]. Another approach has been the creation of soluble recombinant receptor fragments that can be individually tested for ligand binding [41]. These studies have also been aided by the discovery of the ~39 kDa receptor-associated protein (RAP) that has high affinity for LRP (for review see [42]). RAP is normally an ER resident protein that serves as a molecular chaperone for members of the LDL receptor family to prevent premature binding of ligands and aid in their proper folding. Purified RAP is an excellent pharmacological tool because its exogenous application was found to universally antagonize binding of ligands to LRP as well as other members of the LDL receptor family. The majority of LRP ligands, including RAP, have been shown to bind to domains II and IV with equal affinity. No ligand has been demonstrated to bind domain I and only RAP and apoE were found to bind Domain III [40,43,44]. LRP was initially identified as a receptor for activated alpha-2-macroglobulin (α2M) [45]. Since then, LRP has been shown to bind and endocytose over 30 structurally and functionally diverse ligands. The function of these ligands can be divided into several classes, including lipoprotein metabolism, proteinases, proteinase-inhibitor complexes, blood coagulation factors, growth factors, extracellular matrix proteins, chaperones, and bacteria/viral proteins. Numerous ligands may bind LRP by interactions with either a combination of repeats within a single ligand-binding domain or several repeats from separate ligand-binding domains [46].

The 100 amino acid cytoplasmic tail of LRP contains two NPXY motifs, one YXXØ motif, and two di-leucine motifs. A unique feature of LRP is its rapid rate of endocytosis, with half of the receptors at the cell surface able to internalize within 30 seconds (t_1/2 _< 0.5 min) [47]. Other members of the LDL receptor family endocytose at much slower rates, e.g., megalin has a t_1/2 _~ 4.8 min and the apoER2 has a t_1/2 _~ 8.1 min [48]. Using site directed mutagenesis, Li *et al*. (2000) defined the YXXL motif and distal di-leucine repeat as the major endocytic motifs within the LRP tail. Although the ability of LRP to rapidly endocytose a wide variety of ligands suggests a primary function as a cargo transporter, several studies have found that the LRP cytoplasmic domain interacts with proteins involved in cell signaling, axonal transport, and glutamate receptor scaffolding. These adaptor proteins include disabled-1 (Dab1), FE65, JIP-1 and 2, and PSD-95 [49,50]. These findings indicate that LRP may have dual roles as both a signal transduction receptor and a major cargo transport receptor. LRP may also function in synaptic plasticity and memory via association with tissue-type plasminogen activator (tPA) [51], by influencing calcium influx via NMDA receptors [52], or interactions with ApoE and KPI-containing APP in the dentate gyrus [53].

## LRP and Alzheimer's disease

Since LRP is a neuronal receptor for apoE, a well-known AD risk factor, LRP was investigated for its significance in AD pathology. From these studies, LRP has been linked to AD in several ways. First, LRP mediates the clearance of Aβ *in vitro *either by binding to Aβ itself or Aβ complexed to apoE, activated α_2_M, or lactoferrin [54-57]. Second, LRP and its ligands are found in amyloid plaques in AD brains and also in fibrillar amyloid plaques in a mouse model of AD [58-60]. Finally, several polymorphisms within the LRP gene on chromosome 12 have been associated with AD: a 5' tetranucleotide repeat, a single base pair change within exon 3 (*C766T*), and a weakly protective polymorphism in exon 6 [61-63]. It must be stated however, that a number of recent papers have discounted an association between the 5' tetranucleotide repeat of LRP and AD [64-66]. Conversely, the *C*→*T *change in exon 3 has been confirmed in additional studies and in distinct ethnic groups [65,67-69]. Although this silent polymorphism does not affect protein structure, an analysis of AD patients revealed that carriers of the *T *allele had a later age of onset than non-carriers. AD cases with *C/T *or *T/T *genotypes also had significantly higher levels of cortical LRP compared to carriers of the *C/C *genotype, indicating a possible protective effect of higher levels of LRP and/or the *T *allele [70]. Additional studies may be required as this polymorphism has been criticized as being only weakly correlated with AD [71]. Overall, these findings suggest that LRP could play a role in the development of AD pathology.

## Interaction between LRP and APP

Although it is possible that LRP plays a role in the clearance of Aβ, it also alters the metabolism of Aβ via extracellular and intracellular interactions with the Aβ precursor, APP. Several studies have indicated that APP processing to Aβ is modified by LRP expression. In 1995, Kounnas *et al*. reported that LRP binds and internalizes secreted sAPPα, which contains a KPI domain. Soon after, it was demonstrated that cell surface KPI-containing APP complexed with epidermal growth factor binding protein (EGFBP) is internalized by LRP [72]. This internalization of APP was inhibited by the LRP antagonist, RAP, indicating that cell surface and secreted APP are degraded by a mutual pathway that requires LRP.

An intracellular interaction also exists between LRP and non-KPI containing APP through the cytoplasmic adaptor protein, FE65. FE65 contains a WW domain and two phosphotyrosine binding domains (PTB1 and PTB2), similar to the adaptor protein Shc. The PTB domains of FE65 specifically bind ψXNPXpY (where ψ is a hydrophobic residue and pY is phosphotyrosine) motifs within receptor tails [73]. APP binding to FE65 is not dependent on phosphorylation, but can be abolished by mutation of the first tyrosine within the YENPTY motif of APP [74]. Pull down experiments demonstrated that the amino-terminal PTB1 of FE65 binds to LRP and the carboxyl-terminal PTB2 of FE65 binds APP [50], suggesting that FE65 could act as an adaptor to complex these two proteins. A cytoplasmic interaction between APP and LRP, bridged by FE65, could further strengthen the association between LRP and KPI-containing forms of APP and also account for an association between non-KPI containing APP and LRP. These interactions between APP and LRP at cell surface and in the Golgi apparatus have been substantiated with cell surface biotinylation, co-immunoprecipitation, and fluorescence resonance energy transfer (FRET) experiments in cells overexpressing APP, LRP and FE65 [75,76].

To determine if disrupting the interaction between LRP and APP could influence the processing of APP to Aβ, Ulery *et al*. (2000) antagonized the extracellular interaction between cell surface APP and LRP with RAP. Cells expressing APP751 were incubated with RAP for five days. Remarkably, long-term treatment of cells with RAP caused an increase in cell surface APP and a decrease in Aβ production. In the same study, co-transfection of APP and LRP in LRP-deficient cells, led to a ~3-fold increase in Aβ levels in the media compared to media from cells transfected with APP alone. These data demonstrate that LRP expression can influence APP processing to Aβ, possibly via an extracellular interaction between LRP and APP.

In a study utilizing LRP+/- or LRP-/- mouse fibroblasts expressing APP751, Pietzrik *et al*. (2002) demonstrated that cells endogenously expressing LRP or transfected with an LRP C-terminal fragment have increased Aβ levels and decreased sAPPα levels compared to LRP-null cells. These studies indicate that the cytoplasmic domain of LRP alone is sufficient for its effect on APP processing. In the same study, it was demonstrated that cells expressing LRP have a higher ratio of intracellular to cell surface APP compared to LRP-null cells, suggesting that the internalization rate of APP is enhanced with LRP expression. Interestingly, expression of an LRP C-terminal fragment bearing a Y→A mutation within the distal NPXY motif did not decrease sAPPα levels. Since this tyrosine is also important for LRP endocytosis [47], it is possible that mutation of this residue influenced the endocytosis of this LRP fragment, which would also affect APP endocytosis and processing. Studies in our laboratory found that mutations within this distal NPXY motif of LRP as well as the leucine within the YXXL endocytic motif increased cell surface levels of LRP and also resulted in an accumulation cell surface APP [77]. Interestingly, we found that only when both LRP and APP were overexpressed together that there was a net accumulation of extracellular Aβ. When LRP alone was overexpressed, endogenous Aβ levels in the media were lower likely due to the ability of LRP to bind and endocytose Aβ.

Ye *et al*. (2005) recently reported that application of ApoE4 to cells expressing KPI-lacking APP increases APP endocytosis and Aβ levels [78]. This effect was abolished when cells were co-incubated with RAP or when expression of LRP was reduced using small interference RNA. These results suggest that the binding of ApoE to LRP cause levels of Aβ to increase. Although this study provides an interesting link between the pathogenic allele of ApoE, LRP, and APP endocytosis and processing, it is unclear how the binding of ApoE4 to LRP influences APP processing. Future studies should determine if ApoE binding to LRP alters LRP endocytosis, localization, or its ability to interact with APP. Since these studies were performed with APP which lacks an extracellular interaction with LRP, it is possible that ApoE4 binding would enhance an intracellular interaction between LRP and APP.

Our laboratory has also demonstrated a significant link between LRP expression and Aβ levels *in vivo *[79]. Expression of a functional LRP minireceptor in neurons of an amyloid mouse model of AD was associated with an increase in soluble Aβ levels and memory deficits in aged mice. These changes in APP trafficking and processing appear to be linked to the rapid rate of LRP endocytosis. Altogether these findings indicate that interactions between LRP and APP have the ability to modulate Aβ levels.

Recent findings that LRP interacts with presenilin 1 and BACE and is a substrate for γ-secretase and β-secretase [80-82] suggest that the alterations in APP processing by LRP could be more complex than originally considered. LRP may influence APP access to secretases through interactions with the secretases themselves or by changing the compartmentalization of APP. In the case of γ-secretase activity, LRP C-terminal fragments may compete with APP as a substrate for cleavage [82]. In the case of β-secretase activity, LRP may co-operatively aid interactions between APP and BACE possibly in lipid raft domains [80,83]. The recent generation of a mouse that selectively lacks LRP in differentiated neurons may provide a useful model to further analyze the affect of LRP on amyloid deposition [84].

## LRP1B

LRP1B was first characterized in 2000 as a novel LDL receptor family member with extensive homology to LRP [85]. LRP1B shares 59% amino acid identity with LRP. The overall structure of LRP1B is like LRP with similar spacing of its 32 cysteine-rich ligand binding repeats into four clusters of putative ligand binding domains, eight EGF-precursor domains and two NPXY motifs within its cytoplasmic tail. There are two major structural differences between LRP and LRP1B. LRP1B contains one additional ligand binding repeat within ligand binding domain IV and also has a unique 33 amino acid sequence within in its cytoplasmic tail [85,86] (See Fig. 2).

LRP1B was initially named LRP-deleted in tumors (LRP-DIT) because in a study of non-small cell lung cancer cell lines (NSCLC) the *LRP1b *gene was deleted or inactivated in 40% of the cell lines [85]. Since then, inactivation of the *LRP1b *gene has also been described in grade 3 (G3) urothelial cancers [87] and esophageal squamous cell carcinomas [88]. Due to its loss of function in cancer cell lines, it is hypothesized that LRP1B acts as a tumor suppressor. Additionally, the expression pattern of LRP1B suggests that it may have an important function in the brain. Tissue expression analysis established that LRP1B was expressed primarily in the brain, thyroid and salivary gland [86] and *in situ *hybridization of tissue sections determined that LRP1B mRNA expression in the brain was highest in the dentate gyrus of the hippocampus and ventral to the fourth ventricle [89].

In order to determine the trafficking and function of LRP1B, our laboratory created a minireceptor consisting of its fourth putative ligand binding domain, full transmembrane domain, and cytoplasmic tail [86]. This LRP1B minireceptor, designated mLRP1B4, contains both of the main structural differences between LRP and LR1B – an extra ligand binding repeat and a cytoplasmic 33 amino acid repeat. RAP binds both mLRP1B4 and the analogous LRP minireceptor (mLRP4). Several other LRP ligands also bind LRP1B, including complexes of urokinase plasminogen activator and plasminogen activator inhibitor type-1 [86]. Utilizing ^125^I-labeled RAP, Liu *et al*. (2001) measured the endocytosis rate of the LRP1B minireceptor. LRP1B exhibits a much slower rate of endocytosis (t_1/2 _> 10 min) compared to LRP (t_1/2 _< 0.5 min) [86], which may influence the cellular distribution and catabolism of ligands [86,90].

Since LRP1B shares several ligands with LRP, we sought to determine whether LRP1B could also interact with APP. If the fast endocytosis rate of LRP is responsible for facilitating APP processing to Aβ [91,92], we hypothesized that an interaction between APP and LRP1B, which has a much slower rate of endocytosis, would lead to decreased Aβ production. Using an LRP1B minireceptor, we found that mLRP1B4 and APP form an immunoprecipitable complex [93]. Furthermore, mLRP1B4 bound and facilitated the degradation of a soluble isoform of APP containing a Kunitz proteinase inhibitor (KPI) domain, but not soluble APP lacking a KPI domain. A functional consequence of mLRP1B4 expression was a significant accumulation of APP at the cell surface, which is likely related to the slow endocytosis rate of LRP1B. More importantly, mLRP1B4 expressing cells that accumulated cell surface APP produced less Aβ and secreted more soluble APP. Consistent with our finding of decreased Aβ levels, mLRP1B4 transfected cells also had 40% less β-CTF to full-length APP compared to empty vector transfected cells [93].

To determine whether β-secretase processing was a limiting factor to the production of Aβ in mLRP1B4 expressing cells, we transiently transfected the β-cleaved APP fragment, C99, into mLRP1B4 or empty vector transfected cells. We still detected less Aβ in the media of mLRP1B4 cells compared to empty vector transfected cells, indicating that alterations of APP/C99 trafficking rather than changes in β-secretase activity likely contributed to the decreased levels of Aβ found in mLRP1B4 expressing cells [93].

Using an antibody against the C-terminus of LRP1B, we confirmed the expression LRP1B at the protein level in the cortex, hippocampus, and cerebellum. Interestingly, we detected the highest levels of LRP1B in the cerebellum which is a region that is relatively unaffected in AD [93]. Future studies are still needed to address if LRP1B levels are altered in human brain during normal and pathological states. Since these studies suggest its expression may decrease the extracellular release of Aβ, examination of the regulation of LRP1B may have important applications to AD therapy.

## SorLA/LR11

SorLA/LR11 was first described in 1996 as a ~250 kDa receptor containing 11 putative ligand-binding complement-type repeats, 5 YWTD domains, and a vacuolar protein sorting 10 protein (vps10p) domain, which is homologous to a yeast receptor that transports proteins between the late Golgi and a prevacuolar endosome-like compartment [94]. Abundant mRNA expression of sorLA/LR11 was found in human brain, spinal cord, and testis [95]. The functions of SorLA/LR11 are not entirely known. It shares structural and functional similarities with the LDL receptor family in its ability to bind and internalize RAP, ApoE, and lipoprotein lipase [95,96]; however, its endocytosis rate is much slower than LRP. A chimeric receptor of the cytoplasmic and transmembrane domains of SorLA/LR11 endocytosed only 60% of bound ligand after 15 min of incubation at 37°C [96]. Since SorLA/LR11 is also considered to be part of the family of VPS10 domain containing receptors, its main role may be to chaperone proteins as an intracellular sorting receptor. SorLA/LR11 does not appear to play an important role in development since SorLA/LR11 receptor-deficient mice were viable and fertile [97,98].

It was hypothesized that SorLA/LR11 expression may play a preventative role in AD dementia because SorLA/LR11 transcripts were down-regulated in lymphoblasts from AD patients. Also less SorLA/LR11 protein was found in neurons from AD brains by immunocytochemistry and Western blotting [99]. To determine the relevance of SorLA/LR11 expression for Aβ processing, Andersen *et al*. (2005) examined if SorLA/LR11 could interact with APP and affect its cellular localization [97]. Using several methods, including surface plasmon resonance analysis, immunocytochemistry and co-immunoprecipitation, they showed that SorLA/LR11 and APP interact. Further studies elucidated that APP interacts with the extracellular cluster of ligand-binding complement-type repeats in SorLA/LR11 similar to APP binding to the ligand-binding repeats in LRP [77,100]. Unlike the interaction between LRP and LRP1B, the KPI domain of APP was not necessary for an extracellular interaction with SorLA/LR11 [100].

Expression of SorLA/LR11 shifted the localization of APP in membrane fractions from the ER and plasma membrane to the *cis*-Golgi and early endosomes [97]. In a neuronal cell line, SorLA/LR11 expression also reduced surface-localized APP and resulted in an accumulation of mature, glycosylated APP [97]. These alterations in APP trafficking were associated with a decrease in APP processing to Aβ. In this study, APP was overexpressed with SorLA/LR11 and levels of full-length APP appeared to be stable. In another study in which APP was not overexpressed, SorLA/LR11 expression also reduced levels of full-length endogenous APP although the authors note that APP mRNA levels were unchanged [101]. Whether APP was overexpressed or not, SorLA/LR11 expression significantly decreased Aβ levels [97,101]. These findings suggest that expression of SorLA/LR11 alters APP trafficking through the secretory pathway, prevents APP trafficking to the cell surface and may influence its turnover. In agreement with the correlative studies in humans and *in vitro *findings, ablation of SorLA/LR11 expression in mice increased endogenous murine Aβ levels in the cerebral cortex by ~30% compared to controls [97].

Although SorLA/LR11 expression altered the trafficking of APP within intracellular compartments, it did not change the endocytosis rate of APP as in the case of LRP [92,102]. Interestingly, SorLA/LR11 was found to interact with BACE and appeared to compete with interactions between APP and BACE in the Golgi apparatus [102]. Additionally, it has been reported that SorLA/LR11 is also proteolytically processed by γ-secretase [103] and proposed to act as a competitive substrate with APP for γ-secretase activity. Altogether these current findings indicate that SorLA/LR11 regulates APP trafficking into discrete intracellular compartments and also influences its interactions with secretases. Decreased levels of SorLA/LR11 as found in AD could result in increased interactions between APP and BACE which would enhance its processing to C99. With less SorLA/LR11 expression, C99 would have a greater chance to be cleaved by γ-secretase resulting in higher levels of Aβ. Since SorLA/LR11 limits APP processing to Aβ it would be important to know how this receptor is regulated. It has been previously demonstrated that the expression and proteolysis of SorLA/LR11 can be enhanced by binding to its ligands, such as the neuropeptide head activator [104]. Other ligands of SorLA/LR11 *e*.*g*. ApoE, or possible ligands *e*.*g*. sAPP, that are linked to AD pathogenesis are potential candidates and should be investigated.

## ApoER2

ApoER2 is primarily known for its role in cortical development and neuronal migration. Together with VLDLR, ApoER2 acts as a co-receptor for Reelin, an important extracellular signaling protein that regulates positioning of cortical neurons [105]. Recently, several studies have suggested that Reelin binding to ApoER2 may also influence synaptic function, learning, and memory in the adult brain. ApoER2 knock-out mice have deficits in learning and memory and hippocampal slice preparations from these mice are deficient in long term potentiation (LTP), a phenomenon associated with synaptic plasticity [106]. The reason for these deficits could be due to the latest finding that ApoER2 interacts extracellularly with the NR1, NR2A and NR2B subunits of NMDA receptors and also associates intracellularly with PSD-95, proteins which both have important roles in synaptic plasticity [107,108]. Also, alternative splicing of the gene encoding ApoER2 results in a 59 amino acid cytoplasmic insert that is selectively upregulated during periods of high activity in mice. The presence of this insert in ApoER2 was necessary for the propagation of LTP by Reelin and also in NMDA receptor phosphorylation after Reelin binding [107].

In addition to its interactions with the NMDA receptor, it has recently been found that ApoER2 can associate with APP through F-spondin, a protein associated with the extracellular matrix [109]. F-spondin expression alone was previously shown to inhibit BACE-dependent APP cleavage and decrease APP β-CTF levels [110]. An interaction between APP and ApoER2 was demonstrated by co-immunoprecipitation of lysates from primary neuronal cultures and COS7 cells. Incubation with F-spondin-containing medium increased this interaction by over 100%, suggesting an important role of F-spondin in the clustering of these proteins [109]. Interestingly concurrent expression of APP, ApoER2 and F-spondin results in an increase in cell surface levels of both APP and ApoER2.

The clustering of ApoER2 and APP with F-spondin alters the processing of both of these cell surface proteins. With increased cell surface levels of ApoER2 and APP, the authors found an increase in secreted ApoER2 and sAPPα and an increase in their CTFs [109]. ApoER2 expression with F-spondin was also associated with reduced Aβ and β-CTF levels, indicating a decrease in APP processing by β-secretase. The ability of F-spondin and ApoER2 to decrease APP processing by β-secretase was inhibited by preincubation of cells with RAP, indicating the extracellular domain of ApoER2 is important for F-spondin and APP interactions [109]. It would be interesting to know if other extracellular ApoER2 ligands, such as ApoE, could also disrupt ApoER2 interactions with APP and alter its influence on APP processing.

Utilizing a well known FE65-dependent APP luciferase transactivation assay [24] in which luciferase transactivation is dependent on FE65 and γ-secretase cleavage of APP, it was found that application of soluble F-spondin decreased luciferase transactivation [109]. This decrease in luciferase transactivation by the APP fragment and the accumulation of APP CTFs with ApoER2 expression suggest that ApoER2 could also influence downstream transcriptional activity of APP. Future studies are needed to address whether these processes could influence synaptic activity and AD pathogenesis.

A novel finding is that like LRP, ApoER2 is proteolytically processed by a metalloproteinase at the cell surface which releases an extracellular soluble fragment, and subsequently by γ-secretase to release an intracellular domain [111]. Binding of ApoER2 ligands, ApoE and α2M, as well as F-spondin to ApoE influences ApoER2 proteolysis. Currently, the function of these processed fragments is unknown. Subsequently, it will be essential to determine if the proteolytic processing of ApoER2 could alter its interactions with APP and NMDA receptors and influence Aβ levels and/or synaptic activity.

## Conclusion

**Figure 3:**
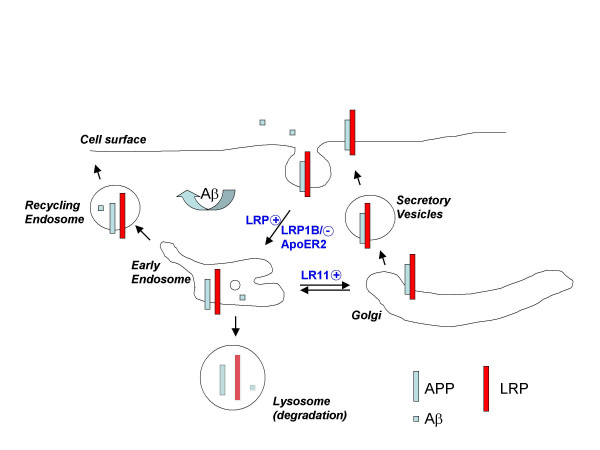
**Model of APP processing pathways mediated by LDL receptor family members. **LRP endocytosis enhances APP endocytosis and processing to Aβ. Due to its slow rate of endocytosis LRP1B retains APP at the cell surface and decreases its processing to Aβ. ApoER2 enhances interactions between APP and F-spondin at the cell surface and also decreases its processing to Aβ. SorLA/LR11 may shuttle APP to the Golgi and prevent its processing by β-secretase in the early endosome, thus decreasing processing to Aβ.

Alterations in APP processing to favor Aβ production and the accumulation of Aβ in the brain are key pathogenic events in AD. A number of proteins, including several members of the LDL receptor family, have been found to interact with APP and regulate its trafficking and processing. In this review, we have discussed several possible mechanisms by which APP trafficking and processing are regulated by LRP, LRP1B, SorLA/LR11 and ApoER2 (Fig. 3). For LRP and LRP1B, the expression and endocytosis of these receptors may have opposing roles in their ability to influence APP endocytosis and thus result in increased Aβ levels with LRP and decreased Aβ levels with LRP1B expression. Expression of SorLA/LR11 alters trafficking of APP to discrete intracellular compartments that result in a decrease in Aβ levels. The uncovering of an interaction between ApoER2, APP, and F-spondin reveals a complex between the extracellular matrix and ApoER2 at the cell surface that can decrease APP processing to Aβ. Although we have focused primarily on the roles of these LDL receptor family members in APP, these receptors are also regulated by alternative splicing and subject to proteolysis that can influence intricate intracellular signaling pathways. Future studies are needed to determine if interactions between these receptors, APP and other ligands or co-receptors can activate downstream signaling cascades that may have ultimately effect the pathogenesis of AD. The regulation of APP processing to Aβ is inherently complex; nonetheless, the discovery that these LDL receptor family members are able to affect its processing is an important step to uncovering new therapies to reduce Aβ and its associated dementia.

## Abbreviations

LDL, low-density lipoprotein; AD, Alzheimer's disease; Aβ, amyloid-β peptide; APP, β-amyloid precursor protein; APLP1, amyloid precursor like proteins-1; APLP2, amyloid precursor like proteins-2; sAPP, soluble APP fragment; sAPPβ, soluble β-cleaved APP fragment; BACE, β-site APP cleaving enzyme; CTF, C-terminal fragment; PNII, protease nexin II; ER, endoplasmic reticulum; CHO, Chinese hamster ovary; LRP, low-density lipoprotein receptor-related protein; VLDLR, very low density lipoprotein receptor; apoER2, apoE receptor 2; sorLA, sortingprotein-related receptor containing LDLR class A repeats; RAP, receptor-associated protein; α2M, alpha-2-macroglobulin; tPA, tissue-type plasminogen activator; FE65L1, FE65-like protein; EGFBP, epidermal growth factor binding protein; mLRP4, LRP minireceptor; mLRP1B4, LRP1B minireceptor; KPI, Kunitz proteinase inhibitor; vps10p, vacuolar protein sorting 10 protein; LTP, long-term potentiation.
